# Whole-Genome Sequencing of *Salmonella enterica* subsp. *enterica* Serovar Cubana Strains Isolated from Agricultural Sources

**DOI:** 10.1128/genomeA.01184-13

**Published:** 2014-01-23

**Authors:** Faiza H. Benahmed, Gopal R. Gopinath, Hua Wang, Junia Jean-Gilles Beaubrun, Christopher Grim, Chorng-Ming Cheng, Michael McClelland, Sherry Ayers, Jason Abbott, Prerak Desai, Jonathan G. Frye, George Weinstock, Thomas S. Hammack, Darcy E. Hanes, Mark A. Rasmussen, Maureen K. Davidson

**Affiliations:** a U.S. Food and Drug Administration, Center for Veterinary Medicine, Division of Animal and Food Microbiology, Laurel, Maryland, USA; b U.S. Food and Drug Administration, Center for Food Safety and Applied Nutrition, Office of Applied Research and Safety Assessment, Division of Virulence Assessment, Laurel, Maryland, USA; c U.S. Food and Drug Administration, Center for Food Safety and Applied Nutrition, Division of Microbiology, College Park, Maryland, USA; d U.S. Food and Drug Administration, Pacific Regional Laboratory Southwest, Irvine, California, USA; e University of California, Department of Microbiology and Molecular Genetics, Irvine, California, USA; f Agricultural Research Service, Bacterial Epidemiology and Antimicrobial Resistance Research Unit, Athens, Georgia, USA; g The Genome Institute, Washington University School of Medicine, St. Louis, Missouri, USA

## Abstract

We report the draft genomes of *Salmonella enterica* subsp. *enterica* serovar Cubana strain CVM42234, isolated from chick feed in 2012, and *S*. Cubana strain 76814, isolated from swine in 2004. The genome sizes are 4,975,046 and 4,936,251 bp, respectively.

## GENOME ANNOUNCEMENT

The presence of bacterial pathogens, such as *Salmonella*, in animal feed is an important route of animal infections and thus is an important risk factor for the human food chain and public health. There are >2,500 serovars of *Salmonella*, and all of them are considered a potential threat to human health (1). *Salmonella enterica* subsp. *enterica* serovar Cubana has been reported in swine that received contaminated feed (2) and in human foods (3). Whole-genome sequencing of *Salmonella* serotypes will allow the discovery of new genes unique to *Salmonella* and will support outbreak investigations.

The *S*. Cubana CVM42234 strain was isolated from chick feed with the Bacteriological Analytical Manual *Salmonella* culture method (4, 5), detected by a quantitative PCR (qPCR) method (6, 7), and serotyped with PCR serotyping (8) and by traditional serological methods according to the CDC protocols (9–11). The serotype antigens are 1, 13, 23, and z29-. Pulsed-field gel electrophoresis (PFGE) was performed according to the CDC methods (12), and the PFGE pattern was JDGX01.0018. *In vitro* antimicrobial susceptibility testing was done according to the standard National Antimicrobial Resistance Monitoring System protocol using the CMV2AGNF panel of antimicrobials (13). *S*. Cubana strain 76814 was tested using a similar microbroth dilution method (Trek Biosystems, Cleveland, OH). Strain CVM42234 was susceptible to all antimicrobials tested, but strain 76814 was resistant to streptomycin, sulfamethoxazole, and tetracycline.

Strain CVM42234 DNA was extracted using the QIAcube (Qiagen, Valencia, CA), the DNA library was constructed according to the Illumina protocol with Nextera XT DNA Sample Prep kit, and it was sequenced using Illumina MiSeq (Illumina, San Diego, CA). CLC bio software version 6.0.1 (Germantown, MD) was used for the trimming and *de novo* assembly of the paired-end reads to 100 contigs. Strain 76814 DNA was isolated with the GenElute isolation kit (Sigma-Aldrich, St. Louis, MO) for bacteria, the library was made by shearing and ligating Illumina sequencing adapters to the genomic DNA, and it was sequenced on HiSeq 2000 (Illumina) then assembled using one Button Velvet (European Bioinformatics Institute, Hinxton, Cambridgeshire United Kingdom) into 247 contigs (172 scaffolds).

Both the CVM42234 and 76814 draft genomes were annotated using RAST (14). The two *S*. Cubana genomes have an average nucleotide identity of 99.5% and reveal many common genetic features, as well as some differences. They both have a multidrug resistance *mdtABCD* cluster, a multiple antibiotic resistance (*mar*) locus, and several multidrug resistance efflux pumps. Loci for resistance to metals, such as arsenic, copper, silver, cobalt, zinc, and mercury, are present. Many flagellar motility genes also are present. CVM42234 contains two discrete clusters of phage proteins. A conjugative plasmid and a small plasmid bearing a mercury resistance locus were identified from the 76814 genome assembly. The presence of many antimicrobial resistance genes and strain-specific mobile elements indicate that *S*. Cubana may exhibit typical heterogeneity based on host and geographical factors as seen in many other pathogenic *Salmonella* serovars. The annotations of both genomes are publically available at RAST.

### Nucleotide sequence accession numbers.

The draft genome sequences for these two *Salmonella* serovar Cubana strains have been deposited at DDBJ/EMBL/GenBank under accession no. ATEU00000000 and AZGR00000000.
